# The extraordinary metabolism of vitamin D

**DOI:** 10.7554/eLife.77539

**Published:** 2022-03-08

**Authors:** Carol L Wagner, Bruce W Hollis

**Affiliations:** 1 Division of Neonatology, Shawn Jenkins Children’s Hospital Charleston United States; 2 Darby Children’s Research Institute, Medical University of South Carolina Charleston United States

**Keywords:** placenta, Vitamin D, fetal programming, epigenetics, transcriptomics, Human

## Abstract

The placenta plays an important role in how vitamin D is metabolized and supplied to the fetus.

**Related research article** Ashley B, Simner C, Manousopoulou A, Jenkinson C, Hey F, Frost JM, Rezwan FI, White CH, Lofthouse E, Hyde E, Cooke L, Barton S, Mahon P, Curtis EM, Moon RJ, Crozier SR, Inskip HM, Godfrey KM, Holloway JW, Cooper C, Jones KS, Lewis RM, Hewison M, Garbis SD, Branco MR, Harvey NC, Cleal JK. 2022. Placental uptake and metabolism of 25(OH)vitamin D determines its activity within the fetoplacental unit. *eLife* **11**:e71094. doi: 10.7554/eLife.71094.

Vitamin D helps the intestine to absorb calcium and other minerals that the body needs, and provides support to the immune system. To carry out these roles, vitamin D must be converted into the active hormone calcitriol (also known as 1,25-dihydroxy-vitamin D). First, vitamin D is metabolized by the liver into a compound called 25(OH)D, which then is broken down into its active form calcitriol, mainly in the kidneys. This metabolic process is tightly regulated and relies on calcium and various hormones, including calcitriol itself (Pike and Christakos, 2017).

In 1979, it was discovered that the level of calcitriol circulating in the blood is elevated during pregnancy (Kumar et al., 1979). This massive increase occurs at the start of pregnancy following the implantation of the placenta (Hollis et al., 2011). However, how and why the metabolism of vitamin D changes so drastically during the early stages of pregnancy is not fully understood.

It was initially assumed that the rising levels of calcitriol were generated in the maternal kidneys, where the key enzyme that metabolizes vitamin D is located (Kumar et al., 1979). However, further studies discovered that this active hormone could also be produced outside the kidneys, bringing into question where this excess of calcitriol is coming from (Gray et al., 1981).

The growing fetus cannot synthesize its own vitamin D, and relies on the placenta to transfer the metabolite 25(OH)D from the maternal bloodstream. This compound was thought to pass into the fetus by passively diffusing across the placenta (Greer et al., 1984; Hollis and Wagner, 2013). Now, in eLife, Jane Cleal from the University of Southampton and co-workers – including Brogan Ashley and Claire Simner as joint first authors – report that the amount of vitamin D the fetus receives is actually regulated by the placenta actively taking up and breaking down 25(OH)D (Ashley et al., 2022).

The team (who are based in various institutes in the United Kingdom and the United States) used two new model systems to study how vitamin D metabolites are regulated in the placenta. First, they built a perfusion model using a structure from the placenta, and flowed it with fluids that mimic how blood circulates from the maternal bloodstream to the fetus. Using this set-up, Ashley, Simner et al. were able to infuse vitamin D metabolites into the bloodstream on the maternal side of the structure, and track the amount that was transferred to the placenta and fetal circulation. In addition to this, the team employed various commonly used techniques to explore the effect vitamin D had on fragments of placenta tissue grown in the laboratory.

These models led to the identification of a mechanism that actively uptakes 25(OH)D on the maternal-facing side of the placenta. Once inside, 25(OH)D is further metabolized to calcitriol, where it imparts impressive alterations on specific placental genes. This influences the level of 25(OH)D and its metabolites in both the fetal and maternal circulation.

These findings suggest that the metabolism of 25(OH)D by the placenta may contribute to the increased level of calcitriol observed in the maternal bloodstream during pregnancy. However, these metabolic changes can only account for a small portion of the excess calcitriol detected. Indeed, a previous study found that a pregnant woman whose kidneys could not metabolize vitamin D only experienced a small increase in calcitriol, despite the placenta and the kidneys of the fetus functioning normally (Greer et al., 1984). This suggests that the placenta only contributes a marginal amount of the calcitriol found in the blood during pregnancy, with the maternal kidneys producing the large majority of the excess.

Further experiments using the model systems revealed that 25(OH)D altered the expression of genes and proteins involved in cellular pathways which are critical for the placenta’s role in pregnancy. This is in keeping with an earlier study which showed a significant association between maternal vitamin D levels and the expression of two placenta proteins linked to pre-eclampsia, a condition that causes vascular changes, high blood pressure and abnormal kidney function during pregnancy (Schulz et al., 2017).

Ashley, Simner et al. also found that vitamin D induced epigenetic changes that reshaped how the placenta responded to this compound and its metabolites. This is similar to a previous study in which vitamin D supplements provided during pregnancy reduced the epigenetic changes associated with gestational aging (Chen et al., 2020).

Vitamin D may be pivotal to the function of the placenta, thereby affecting both maternal and fetal health. As such, the work by Ashley, Simner et al. raises important questions about the role this compound plays during pregnancy. Initially vitamin D was thought to only be involved in maintaining calcium levels; however, this study and others suggest it is also important for modifying the immune response of the fetus (Mirzakhani et al., 2016; Khatiwada et al., 2021; Zahran et al., 2018).

Various other questions about the metabolism of vitamin D also remain unanswered. For example, how are such high amounts of calcitriol tolerated during pregnancy, including by the fetus, which would normally lead to fatal levels of calcium? And how does the enzyme in the maternal kidneys, which is highly regulated, lose control and produce such ‘toxic’ amounts of calcitriol? Investigating these questions, as well as others, will provide new insights into how vitamin D metabolism is controlled during pregnancy and will further our understanding of its role in optimizing maternal and fetal health.
